# The Impact of Interface Design Element Features on Task Performance in Older Adults: Evidence from Eye-Tracking and EEG Signals

**DOI:** 10.3390/ijerph19159251

**Published:** 2022-07-28

**Authors:** Chengmin Zhou, Fangfang Yuan, Ting Huang, Yurong Zhang, Jake Kaner

**Affiliations:** 1 College of Furnishings and Industrial Design, Nanjing Forestry University, Nanjing 210037, China; yuanfangfang@njfu.edu.cn (F.Y.); tinghuang@njfu.edu.cn (T.H.); yurong220503@163.com (Y.Z.); 2 Jiangsu Co-Innovation Center of Efficient Processing and Utilization of Forest Resources, Nanjing 210037, China; 3 Nottingham School of Art and Design, Nottingham Trent University, Nottingham NG1 4FQ, UK; jake.kaner@ntu.ac.uk

**Keywords:** interface design, user experience, design element features, eye tracking, EEG signals, older persons

## Abstract

It is crucial that the interface design of mobile apps be age-appropriate at this stage of global aging, as the new epidemic has resulted in a higher sense of isolation among older persons. In this study, four typical senior social service mobile applications were chosen to give older persons the ability to complete user login duties. The participants were 16 older adults (7 men and 9 women) aged 55 to 76. Both objective and subjective data, including task completion time, gaze length, pupil diameter changes, EEG wave amplitude changes, and subjective sensations of older persons, were gathered using a combination of eye-movement and EEG signal approaches. The program was created to investigate the effects of interface design aspects on older people’s task performance, including interface layout, interface color, information density, icon size and position, etc. The study’s findings revealed that when the user task completion time and average fixation duration were shorter, the line of sight was more equally distributed, the visual focus was closer to the login button, and the average EEG amplitude of the user changed more, the older adults performed better. The palace layout had a more positive effect on job completion among older individuals when it came to interface layout. In terms of interface color, colored (contrasting) colors should serve to highlight the interface’s essential information points while they can be removed. In terms of interface information density, a low-density level interface design can simplify and lower the cognitive load of task execution for older people. The first level of icons in the interface and their position in the visual center of the interface is the best interface design for older persons in terms of icon size and position. The results of this study have theoretical ramifications for a thorough understanding of the factors influencing older people’s task performance, practical ramifications for the design of older people-centered interfaces, and they contribute to our understanding of the characteristics of older people’s interface interaction behavior.

## 1. Introduction

The world’s population is aging, which is a pressing concern. China, which has the world’s largest old population, is on the verge of becoming an aging society, with 13.5 percent of the population over 65 by 2020 [1]. Furthermore, due to uncontrollable factors such as declining perceptual and cognitive abilities over time, older people have lower usage rates of smart devices such as mobile phones compared to younger people, as smart devices continue to be upgraded and face-to-face communication between people becomes less frequent [2]. Smartphone ownership among seniors (65 and older) is 30%, compared to 86% among those aged 18 to 29 [3]. While being isolated from social life had little negative impact on young people during the spread of COVID-19, the isolation experienced by the older population has become more pronounced due to a lack of infrastructure in smart devices suitable for the elderly population, aggravating their physical and psychological problems [4,5,6,7]. One study found that older people who use mobile internet are 33.1 percent less lonely than those who do not [8,9,10]. Social apps for older persons, which function like virtual universities for older persons and give them access to information about nursing homes, health and wellness, learning about their interests, and interacting with their peer groups, have been developed in response to the need for older people to socialize and find social circles of interest. The interface design of social apps for older persons in China, however, is still in its infancy. This is why a thorough grasp of the challenges of employing smart devices for older adults and their demand for them is critical to improving their present living conditions in the context of an aging society and long-term home isolation.

User experience research is inextricably linked to good interface design. Older people’s task performance is influenced by interface design features, subjective performance, and external environmental distractions, among other factors, and interface design elements are important determinants of user satisfaction and user mood swings. Research on interface design for older people broadly revolves around three types of smart devices: computer web pages [11,12,13], smart home devices [14,15,16,17], and mobile applications [18]. The proper selection and layout of each design element of the interface is vital in order to accomplish both comfort and accuracy of interface interaction for the senior user group. The three features of interface layout, interface colors, interface icon dimensions, and icon choices are examined based on a wide range of literature. Initially, some researchers proposed a new research method based on a combination of hierarchical analysis and gray theory to evaluate the aesthetics of interface element layout design [19]; others have developed a mobile marketing recommendation method that combines aesthetic preference with layout, discovering that including users’ aesthetic preference factors in interface layout design can lead to better results [20]. Gao Runze et al. [21], for example, studied the page layout of shopping websites based on the most popular points of customers’ visual attention and browsing habits. In terms of result innovation, Li, QC et al. [22] looked into the navigation patterns of older adults’ apps and discovered that they preferred content-oriented design patterns; Wu, ZX et al. [23] discovered that older adults preferred text-only layouts over graphic layouts; and Su, XY et al. [24] discovered that older adults cared more about the central area when browsing web pages while paying more attention to the peripheral area during visual search on web pages. By analyzing eye-movement data collected by older individuals when utilizing different mobile learning platform interfaces, Zhang, MM et al. [25] discovered that vertical layout panel design was more effective than horizontal layout panel design. Furthermore, based on interface color research, Zhang, ZZ et al. [26] discovered that by optimizing color semantics through Spearman rank correlation coefficient analysis, user satisfaction of smartphone interface icon color could be improved; Wu, TY et al. [27] analyzed the visual elements of smart kitchen appliance interfaces for the elderly by combining perceptual engineering evaluation and experimental psychology to obtain the elderly’s preference for interface color information and graphics. Backhaus, N et al. [28] and Wu, JF et al. [29] established two control groups for the selection and identification of icons between young and old people, and between normal and cognitively impaired elderly people, respectively, in terms of interface icon ratio and icon selection, with results that showed that older persons prefer the skeuomorph version. It was concluded that the number of icons in one section of the interface design should not exceed 25, and the spacing between elements should be higher than 1/2 an icon for a modest number of icons [30].

All of the aforementioned research examines how one interface design element affects how older users interact with it and ultimately leads to the selection of interface design elements that are most suitable for older users. In contrast, there are many studies that address the impact of multiple variables of design elements on the use of interface design in older age groups. For example, Kalimullah, K. and Sushmitha, D. et al. [31] investigated the user interface design elements (text size, font, color, etc.) of mobile applications that affect the user experience of older adults and discovered that convenience was the primary influencing factor for older adults to use and continue to use them. Tang, XT et al. [32] investigated the impact of design elements such as font size, background color combinations, spacing, and placement of parametric information on the readability of a vital sign monitoring interface, and discovered that high contrast colors improved accuracy. Yu, N [33] investigated the preferences of an older population using three factors: button size, graphic/text ratio, and icon type, and discovered that older adults preferred 20 mm larger buttons, larger text, and larger icons. The number of icons in one section of the interface design should not exceed 25, and the spacing between elements should be higher than half an icon for a modest number of icons [34].

Interface design is primarily used through human visual engagement as a channel for communicating and exchanging information between humans and machines. Consequently, in the numerous studies currently in the literature, in order to assist in improving the aging of the interface design, the majority of researchers have examined the visual aspects of user interaction by measuring the user’s eye movements. Numerous researchers’ findings also support the idea that eye-movement data can be used to monitor a user’s visual motion and assess the user’s level of concentration, cognitive load, and the appropriate task flow and interface layout using information like pupil diameter, eye trajectory patterns, and gaze duration. Researchers like Johannes Zagermann [35] have discovered that changes in user cognition are reflected in changes in pupil diameter, gaze, and sweeping gaze, with pupil diameter being the most sensitive to the estimation of user cognitive load [36,37,38]. The ability of gaze, an oculomotor measure, to effectively gauge user visual tiredness during task performance has also been demonstrated by Evgeniy Abdulin and colleagues [39]. We can draw the conclusion that eye-movement data is a highly reliable and well-established objective assessment metric in interface design research.

On the other hand, the electroencephalogram (EEG) signal, which records the changes in electrical potential during brain activity, is a bioelectric signal produced by the activity of brain nerve cells in the cerebral cortex. Several studies have shown that EEG signals are more reliable than other signals, such as electrocardiography (ECG) and electromyography (EMG) signals, in detecting minor changes in the body, such as concentration levels and emotional shifts [40,41]. The EEG signals generated by the brain can indicate the user’s psychological changes as they interact with various forms of material (e.g., computer web pages, smart product interfaces, mobile apps, etc.) [42]. Some researchers believe that EEG signals can be used to control and operate virtual home appliances [43], while others want to use EEG signals to investigate the impact of color on interface design [44,45], whilst others want to use EEG signals to analyze user aesthetic preferences in interface layout [46,47]. Therefore, studying user behavior during interface interaction using physiological data, such as eye movement data or EEG signals, can produce the best research results and design approaches.

According to the aforementioned research, eye-movement-related indicators are more developed and widespread in the study of interface design for older persons, whereas EEG signals are rarely employed as an assessment indicator, and the use of combined eye-movement and EEG signals is even less prevalent. Moreover, the current research on the aging-friendly interface of mobile applications is limited because the core users of smart devices such as smart home products, shopping websites, and social software are young people, and older people are rarely the focus group of smart device developers, which means that older people are still unable to use smart devices in the real world [48], according to the findings. The use of smart devices and social interaction among the elder population can be increased by investigating and comprehending their demands and behavioral traits utilizing real-world interfaces. The research hypotheses are as follows: (1) changes in interface design elements, such as interface layout, interface color, information density, icon size and position, etc., significantly influence older people’s task performance by appealing to their eye-based visual approach; (2) eye-movement indicators and EEG signal indicators reflect the impact of interface design elements on older people’s task performance.

In summary, in order to capture the visual activity characteristics of older people during the task execution of visual search and login for four representative social service apps for older persons, this paper proposes a method based on a combination of user eye-movement signal data (i.e., task completion time, average fixation duration, average pupil diameter, and eye-tracking hotspots, etc.) and EEG data. To test older people’s task performance, the data was gathered and processed by gathering eye-movement and EEG signals from them. This was followed by analysis using a variety of techniques, including one-way analysis and repeated measures analysis of variance. The arrangement of the information, the color of the interface, and the size and placement of the login icons all affect how quickly the user completes the activity. This study demonstrates the viability of employing eye-movement and EEG amplitude metrics to analyze user interface interaction design as well as the extent to which interface design aspects affect the effectiveness of task execution for older users. The findings of this study not only contribute to a more thorough analysis of older people’s task execution behaviors, but they also offer tactical recommendations for improving the user interfaces of these four social service apps for older people, which will have a positive influence on the future advancement of interface design for older users and accessibility.

## 2. Materials and Methods

### 2.1. Experimental Participants

Experimental subjects were recruited from three nursing homes in Nanjing, China. The members of our team contacted the nursing home staff by phone, and the staff randomly picked 16 eligible individuals based on our team’s specific needs. All of the subjects were free of color blindness and color weakness, were right-handed, had no physical disabilities, had natural or corrected visual acuity of more than 1.0, and had some schooling to read and write. Participants were required to have healthy eyes and no excessively drooping eyelids to conceal them in order for the oculomotor data to be collected. Seven men (mean standard deviation = 60.57 ± 6.58 years) and nine women (mean standard deviation = 62.33 ± 8.28 years) were among the 16 participants, who ranged in age from 55 to 76 years (mean standard deviation = 61.56 ± 7.39 years). The basic demographic information data for all participants are shown in Table 1. The Ethics Committee of Nanjing Forestry University’s Science and Technology Division gave their approval to the study protocol (Jiangsu Province, China). Before engaging in the experiment, all participants read and signed a consent form, and at the conclusion of the experiment, they were given some experimental recompense.

### 2.2. Experimental Equipment and Environment

The equipment selected for this experiment included the Ergo LAB human–computer environment synchronization cloud platform, the Semi-Dry wearable wireless EEG measurement system, the Tobii Pro Fusion eye-tracking device, and the Redmi K30Pro mobile phone as recording devices. The Tobii Pro Fusion eye-tracking device is a new generation of high-performance portable eye-tracking devices from Tobii Pro, equipped with dual eye-tracking sensors and dual tracking modes (bright and dark pupil), with a sampling rate of 250 Hz, screen resolution of 2400 × 1080 pixels, point-of-view position accuracy of 0.3${}^{\circ }$ and a latency of <13 ms. The Semi-Dry system is a compact portable EEG system that records 8–64 EEG channels in real time; in this experiment, 16 EEG channels were recorded in real time. The entire experimental environment is an air-conditioned, controlled indoor space with good temperature and humidity, good lighting, and no noise. Participants were tested in a natural sitting position, with a soft and stable seat, and the distance between their eyes and the screen was approximately 50–80 cm.

### 2.3. Experimental Stimulus Materials

**Figure 1:**
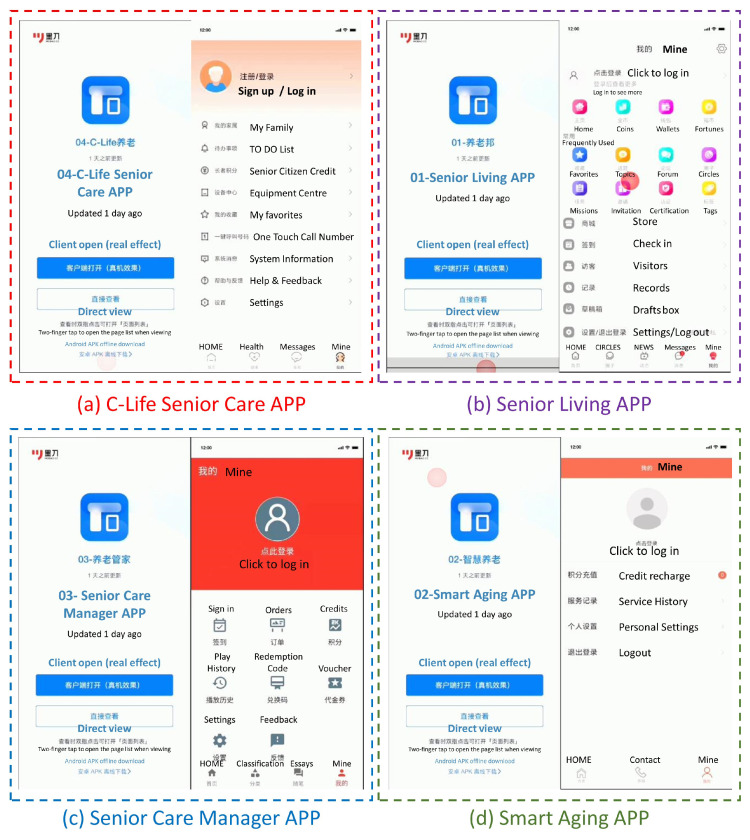
Experimental stimulus material: the login interface of the four apps.

The goal of this study is to see how different interface design features in a senior care service application affect how people utilize the aging-friendly APP for older persons. As a result, the focus of this article is on the functionality and homepage design characteristics of aging-friendly APP items on the Android application mall in the Redmi K30Pro mobile phone. To achieve the greatest degree of visual stimulation during the experiment and monitor the user’s visual changes, 10 candidates for aging APP were initially selected, based on the APP information framework, interface layout characteristics, main color characteristics, and interface information density, among other factors. According to various factors, 17 pages of the first-level interface of four apps with significant differences were chosen as experimental stimulation materials, with Figure 1a representing the “C-Life Senior Care APP”, Figure 1b representing the “Senior Living APP”, Figure 1c representing the “Senior Care Manager APP”, and Figure 1d representing the “Smart Aging APP”. The data collection of visual search tasks for older users is carried out in the experiment for these four common mobile terminal goods for older persons.

This study looked at four elderly-friendly mobile goods to see which interface design features are most appropriate for them. In terms of page layout, the login screens of C-Life Senior Care APP and Smart Aging APP are multi-column layout; the login screen of Senior Living APP is irregular grid layout; and the login screen of Senior Care Manager APP is palace format interface layout. The difference of the Apps by the position of color application is: the login screen of C-life Senior Care APP has a gradient orange background and colored login icons from top to bottom; the login screen of Senior Living APP has a white background + colorless login icons + colored secondary icons; the login screen of Senior Care Manager APP has a high contrast color background + colorless login icons; and the login screen of Smart Care Manager APP has a high contrast color background + colorless login icons. The login screen of Senior Care Manager APP has a high contrast color background + colorless login icon; the login screen of Smart Aging APP has an orange navigation bar + white background + colorless login icon. From dense to sparse, according to the level of information density: Smart Aging APP > Senior Living APP > C-Life Senior Care APP > senior care manager APP > Arrangement of colors: Senior Living APP has a green background with colorful aids; Senior Care Manager APP has a high-saturation solid color; Smart Aging APP has highly saturated contrasting colors; C-life Senior Care APP has a low saturation color gradient.

### 2.4. Experimental Procedure

**Figure 2:**
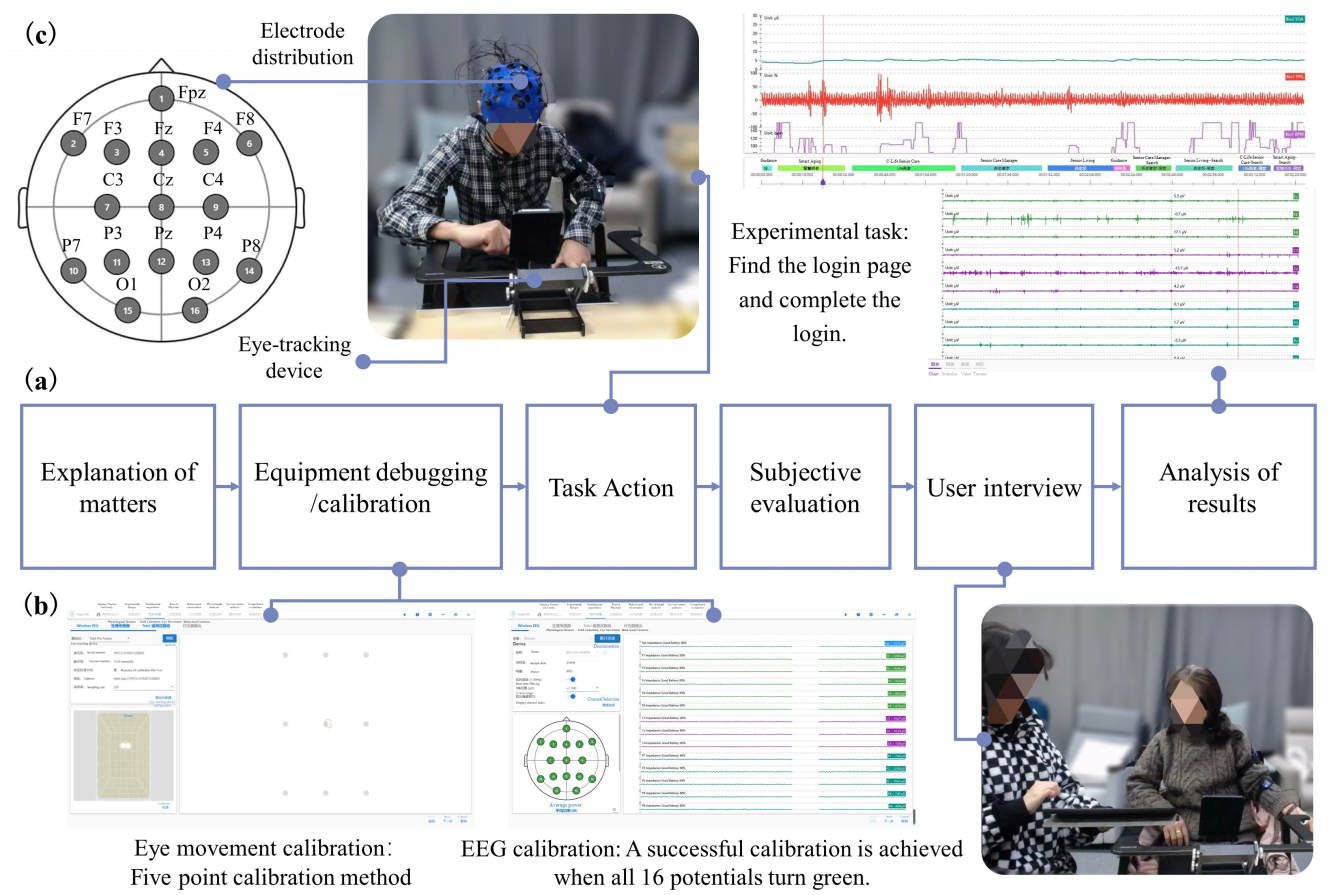
Experimental procedure for EEG recording and eye tracking: (**a**) The overall process of the experiment; (**b**) Instrument adjustment and calibration; (**c**) User experiment diagram.

The experiment was designed to collect and record eye movement changes and brain waves evoked by visual stimuli using different mobile applications in older adults. The experimental procedure is described as follows: Before the experiment started, the subjects entered the lab when the procedure and precautions to be taken were explained. The subject then started to debug the equipment and software, and presented the first-level interface prototypes of four typical age-appropriate mobile products on the screen of the Redmi K30Pro mobile phone. The brightness and color temperature of the screen were ensured to be consistent before the experiment started. Afterwards, the EEG cap and the oculography were calibrated separately. A successful calibration was considered when all electrode points of the EEG cap turned from red to green, the oculography was calibrated using the five-point method of eye data, and then the experiment was ready to start. The subjects began with a 10-min sample familiarization exercise to minimize performance errors due to inexperience. Afterwards, 16 subjects completed the “search for content of interest” task in each of the four apps, and as the main functions of the old and old mobile products were different, but each product had to have a personal login interface to facilitate personalized function pushing, the search task was set to “login”. The main participant was instructed to “search for and click on the login button” to understand the participant’s experience of the interaction process. Throughout the experiment, if the subject took a long time, there was no need to prompt him/her and he/she could just record it as it was. There was a 5–10 min break after each set of APP interface tests were completed with a user interview, where the main subject was asked about the user’s understanding and operational behavior in response to their performance while browsing. The subjects were also asked to choose the APP with the highest satisfaction and to rate the experience of using each of the 4 APPs. To end the experiment and collate the data, the experimental flow is shown in Figure 2.

## 3. Results

### 3.1. Eye Movement Data Analysis

Because people primarily acquire information through visual perception, eye movement analysis is a useful method for processing visual data [49]. By evaluating the recorded data, the oculomotor, as a device for recording eye movements, can explore the relationship between eye movements and human mental functions. The amount of time it takes all participants to do the assignment is referred to as the task completion time. The task is completed faster when the goals are well-defined. The overall amount of time the user spends staring at the task is known as the average fixation duration. The average fixation time will increase if the interface information is more challenging to recognize or if the user is more engaged. The average pupil diameter is the change in pupil diameter over the course of the user’s task. The pupil diameter will grow as the user’s cognitive burden does. The hotspot is the user’s region of attention in the interface, and the eye trajectory is the user’s sweeping route when carrying out a job. In this study, task completion time, mean fixation duration, and mean pupil diameter were utilized to gauge participants’ task performance and cognitive load. Eye trajectory and hotspot maps were employed to track eye movement and vision.

#### 3.1.1. Task Completion Time

**Figure 3:**
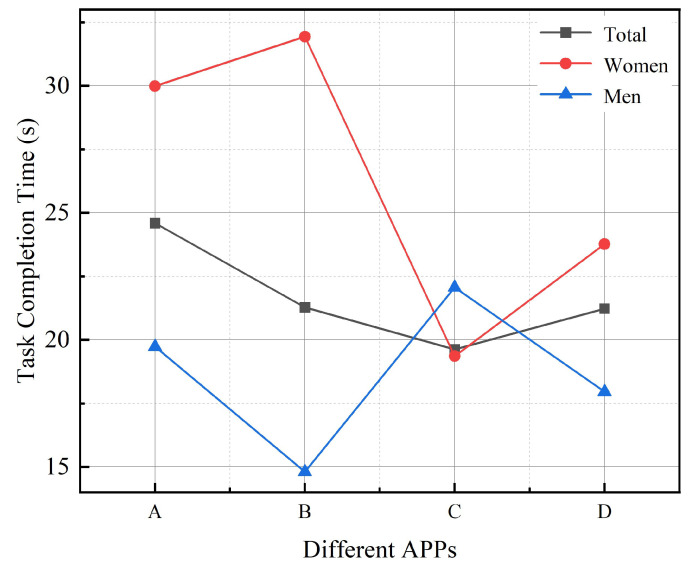
Line chart of participants’ task completion time in different Apps: A refers to C-life Senior Care APP; B refers to Senior Living APP; C refers to Senior Care Manager APP; D refers to Smart Aging APP.

Table 2 shows the results of ANOVA for Task Completion Time. It was found that four different apps had no significant effect on the length of completion time for older adults (F = 3.240, *p* = 0.093 > 0.05). The shortest time was the Senior Care Manager APP (20.55 ± 18.21) and the longest time was the C-Life Senior Care APP (25.50 ± 25.50). Senior Care APP (25.50 ± 15.81). Based on the results of the analyzed and processed data, the relationship between the four different apps and the task completion time was derived, as shown in Figure 3.

#### 3.1.2. Average Fixation Duration

**Figure 4:**
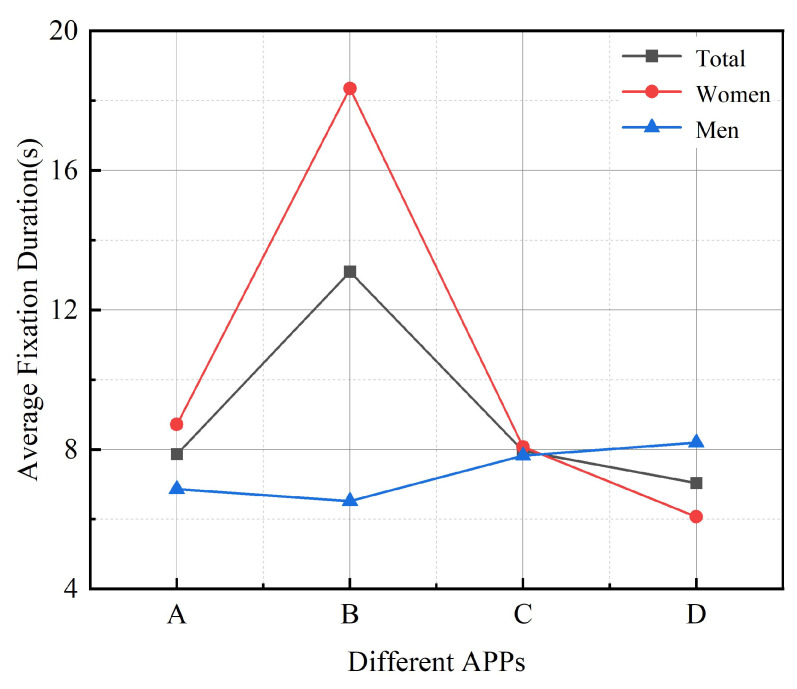
Line graph of the average fixation duration time of participants in different Apps: A refers to C-life Senior Care APP; B refers to Senior Living APP; C refers to Senior Care Manager APP; D refers to Smart Aging APP.

Gaze is the dwell of the human eye while observing a target, and most of the information acquired by the user’s eye is processed and extracted while gazing. The average duration duration reflects the processing time of the target information in the region [50]. An interface with complex information takes longer for the user to process and extract, and the duration of the duration becomes longer. Thus, a longer sustained duration time indicates more difficult user task execution. During the experiment, the eye-tracking device recorded the average duration of older people during the “login” task using four different apps. Table 3 shows the ANOVA results for Average Gaze Time. The study found a significant effect of the four different apps on the duration time of the older person interface (F = 1.667, *p* = 0.007 < 0.05). The average duration was from the longest to the shortest: where Senior Living APP (7.369.11) had the longest average duration and Smart Aging APP (4.845.97) had the shortest average fixation duration, while the average fixation duration of the two apps, Senior Care Manager APP (6.476.42) and C-Life Senior Care APP (6.396.33), did not differ significantly. Based on the results of the analyzed and processed data, the relationship between the four different Apps and the mean fixation duration of older adult was derived, as shown in Figure 4.

This shows that: (1) the relationship between different interface design factors and the average fixation duration of older people is not linear, but has a significant effect, and the average fixation duration of users varies with different interface design factors. For example, the higher the information density level, the smaller the ratio of icons to interface and the uneven distribution of interface colors, the more negative the effect of interface design on task performance of older adults; (2) there is a difference between male and female groups, except for the average fixation duration in Smart Aging APP, the average fixation duration of the female group is much higher than that of the male group, which indicates that gender has a significant effect on task performance. There is a significant difference in the effect of gender on task execution, and the more task interference items there are in the task execution process, the more time-sensitive tasks need to be completed.

#### 3.1.3. Mean Pupil Diameter

**Figure 5:**
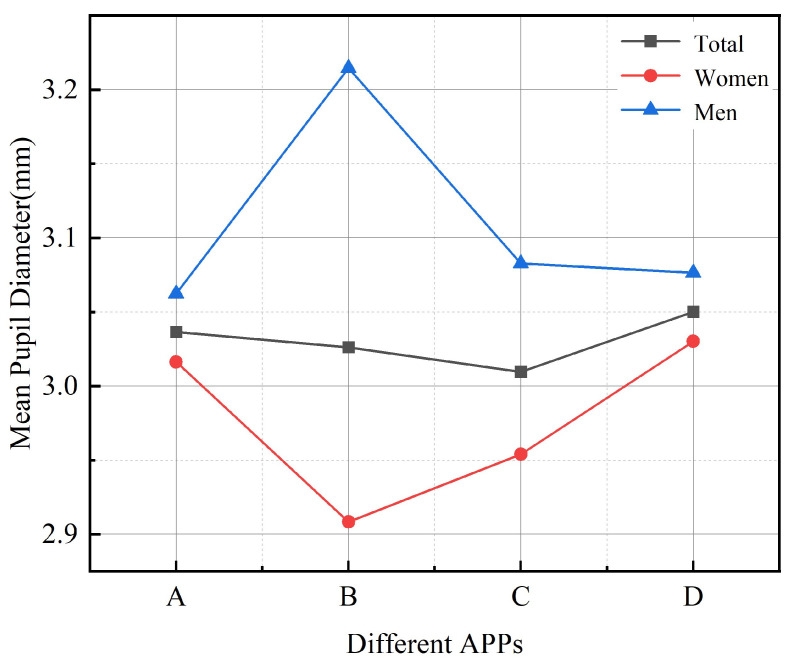
Line graph of mean pupil diameter of participants with different Apps: A refers to C-life Senior Care APP; B refers to Senior Living APP; C refers to Senior Care Manager APP; D refers to Smart Aging APP.

Pupil diameter size can be used as an indication of cognitive load, which is an unconscious reflex. Pupil dilation indicates the subject’s focused attention on observation and is accompanied by the subject’s effortful cognitive processes. Table 4 shows the results of the ANOVA for Mean Pupil Diameter. Four different apps were found to have a significant effect on the change in pupil diameter in older adults (F = 0.931; *p* = 0.008 < 0.05). The mean pupil diameter size ranged from large to small: C-Life Senior Care APP > Senior Care Manager APP > Smart Aging APP > Senior Living APP. The maximum mean pupil diameter was Smart Aging APP (3.04 ± 0.44) and the minimum mean pupil diameter was Senior Living APP (2.46 ± 1.28). The relationship between the four different apps and the average pupil diameter of older people was obtained based on the results of the analyzed and processed data, as shown in Figure 5. It can be seen that there is a significant effect between different interface design factors and the average pupil diameter of older people, and the average pupil diameter of users varies with different interface design factors. For example, the lower the information density level of older people, the less energy and attention they need to use the interface where the login icon occupies the visual center of the interface, and the easier the task execution.

#### 3.1.4. Eye Tracking Diagram

**Figure 6:**
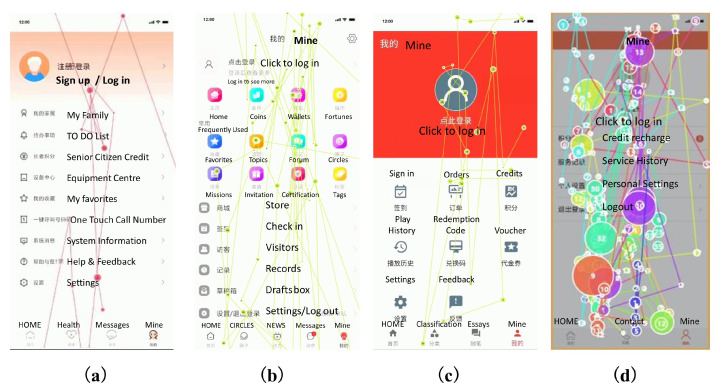
Four product eye movement track maps: (**a**) C-Life Senior Care APP login screen track diagram; (**b**) Senior Living APP login screen track diagram; (**c**) Senior Care Manager APP login screen track diagram; (**d**) Smart Aging APP login screen track diagram.

According to the eye-movement diagram in the eye-movement experiment, we can observe the location of subjects’ first gaze on the mobile product page, which can reflect which elements on the whole page are more visually attractive and the visual search tracking. Usually, the first point of view of users falls in the middle of the page, such as bright color blocks or attractive images, while textual information is usually in a later order of attention because it needs to be reprocessed by the brain, which is based on the characteristics of human visual cognition [51,52]. Observing the user’s eye trajectory during the page login task provides insight into the impact of interface design elements on user task execution. According to Figure 6, the eye-movement chart of the login interface of C-Life Senior Care APP, Senior Living APP, Senior Care Manager APP, and Smart Aging APP are plotted in order from left to right.

The user eye-movement diagrams of the four typical products all fold back in the middle, which corresponds to the general browsing eye movement pattern. A good interface scan path should be clear, well-organized, provide a good user experience, and not obstruct the user’s line of sight excessively. Observing users’ eye movement diagram during page login tasks provides insight into the influence of interface design elements on user task execution. By compiling the eye-movement diagram of all subjects, we found that users had more backward glances in the lower half and the right half of the page, which is related to people’s daily reading habits, where they usually read from left to right and from top to bottom, and when there are other elements on the page that attract the sight, the eye tracking diagram will fold back to continue processing the rest of the stimulus information, thus producing backward glances. From Figure 6a, it can be seen that in the eye tracking diagram of the C-Life Senior Care APP, the user’s back-gaze occurs inconspicuously, and the overall interface is more evenly attractive, which enables users to browse according to their general eye-movement pattern and reading habits, and the user has high freedom of vision. Then, as shown in Figure 6b,c, comparing the eye tracking diagrams of Senior Living and Senior Care Manager Apps, we can see that both users look back very frequently, and due to the difference in information density and page color between them, the return video rate: Senior Living APP > Senior Care Manager APP. It shows that the high contrast color of the page can make the users’ eyes average; the users’ eyes of the Senior Living APP are basically focused on the colored icons, so it has an interference effect on the execution of the task of finding out the location of the login key for older persons. The user eye tracking diagram of the Smart Aging APP, as shown in Figure 6d, shows that it is extremely easy for older people to find the login key. Thus, it can be concluded that an interface layout with a moderate ratio of icons to the interface, a low information density level, and a general line of sight pattern is more suitable for older persons, and it is easier to guide the users to effectively access and process information during the interaction process.

#### 3.1.5. Page Heat Map

**Figure 7:**
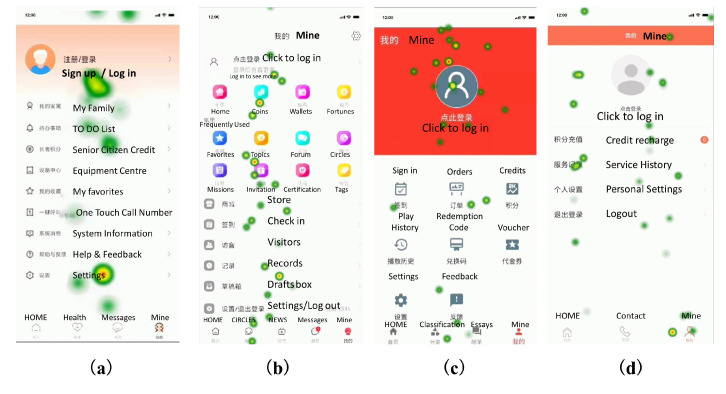
Four product eye movement heat maps: (**a**) C-Life Senior Care APP interface hot spot map; (**b**) Senior Living APP interface hot spot map; (**c**) Senior Care Manager APP interface hot spot map; (**d**) Smart Aging APP interface hot spot map.

The hotspot map reflects the distribution of subjects’ interest points when browsing the interface, and the location of subjects’ attention points when browsing the page can be judged based on the hotspot map. Following the hotspot attention points can let outsiders understand the habits of senior users browsing the product [53]. The hotspot map can be used to visually display the stimulus elements that subjects pay attention to. The gradation of hotspot color from green to yellow to orange to red represents the shortest to longest duration of visual attention, i.e., red represents the longest gaze time and green represents the shortest gaze time, and the hotspot area becomes larger as the gaze time becomes longer. According to Figure 7, in order from left to right, the eye-movement heat map of the login interface of the C-life Senior Care APP, Senior Living APP, Senior Care Manager APP, and Smart Aging APP is shown in order. The following is a detailed explanation of the distribution status of the heat map: (1) As shown in Figure 7a, the red hotspots appearing in the eye-movement hotspot map of C-Life Senior Care App are all in the middle and bottom of the page, and are concentrated on the main navigation bar, which helps guide users to complete the interaction behavior of the main function. However, it also shows that low saturation colors are less attractive to users’ eyes, even if they can complete the interaction tasks on their own, because the interface colors are too mild to attract users’ attention and make the key information on the home page unappreciated. Therefore, adding high saturation color to highlight key information does not only not affect the overall dominance of interactive tasks, but can also attract users’ attention; (2) the home page of Senior Living APP adopts an irregular grid layout, as shown in Figure 7b, users’ attention points are scattered and concentrated on the main navigation bar, so when an irregular grid layout is adopted and the page is rich in color and text information, it is difficult to focus the attention points effectively. (3) As shown in Figure 7c, the user’s eyes are concentrated in the eye-movement hot zone diagram of the Senior Care Manager APP, and the eye-movement hot zone obviously stays in the red color block in the header. This indicates that the high saturated color has stronger visual stimulation for users, higher attraction of sight, and longer time for subjects’ sight to gaze at the page head; (4) The eye-movement hot spot diagram of Smart Aging APP is shown in Figure 7d. On the intuitive interface with card-type design and rich contrast of color, the distribution of users’ sight is more even, which can attract users to shift their sight, and at the same time, it does not overly dominate users’ sight. This indicates that the page elements can attract users’ visual attention, which means that users tend to prefer interactive interface designs with intuitive color contrast and clear and simple pages. Therefore, the color contrast changes to attract the user’s eye shift, and evenly distributed key information can also draw attention to the key information within the page.

### 3.2. EEG Data Analysis

The initial technique for analyzing EEG signals was time-domain analysis. The total reflection of physiological activity at each electrode location in the cerebral cortex or scalp surface during the user’s task performance is called electrode wave amplitude variation. The overall variance in the superimposition of electrode points in each brain area during the user’s performance is known as the wave amplitude variation in brain regions. Different parts of the brain reflect different functional changes in the user; the temporal and frontal regions sense changes in information connected to emotions, while the parietal and occipital regions perceive changes in visual information [54,55].

**Figure 8:**
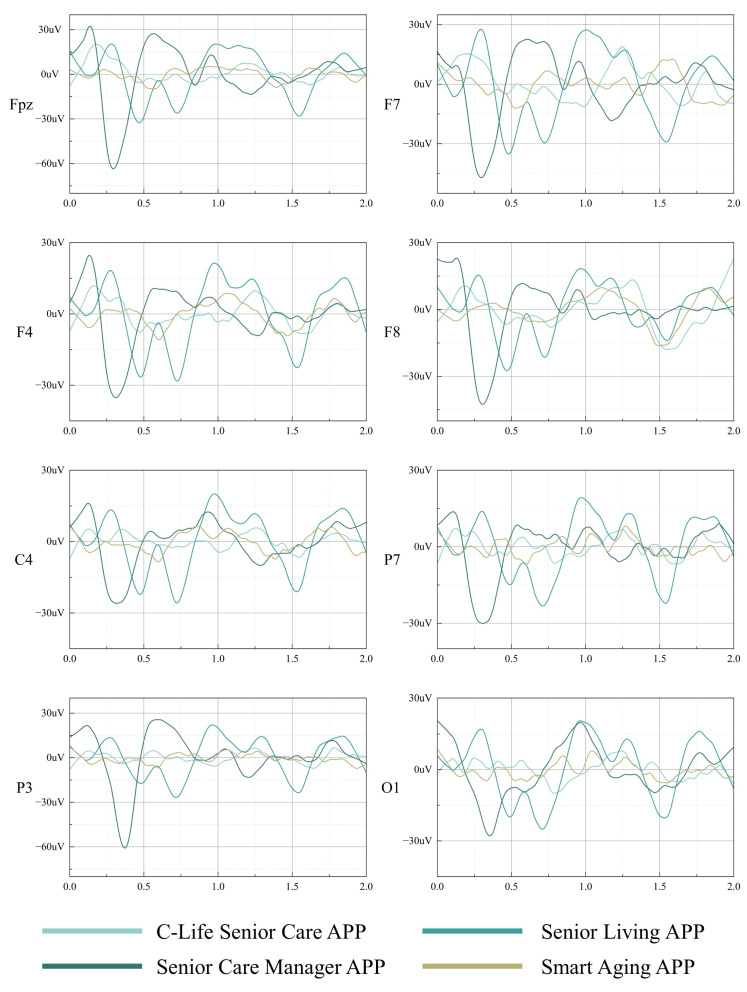
Electrode wave forms of different APP processes for each electrode position.

The raw EEG data is complex, and processing of the raw EEG data is required to turn the experimental data into usable analytical data. Four APP interfaces with different design elements were labeled as A, B, C, and D in the EEG experiment, where A refers to C-Life Senior Care APP, B refers to Senior Living APP, C refers to Senior Care Manager APP, and D refers to Smart Aging APP. The detailed data processing steps are as follows: (1) import electrode coordinates; (2) segmentation according to the whole search task completion time; (3) segmentation according to the whole search task completion time; (4) baseline calibration; (5) calibration of all “A”, “B”, “C”, and “D” for a single subject. “C” and “D” segments of a single subject; (6) averaging and superimposing “A”, “B”, “C”, and “D” segments of a single subject; (7) averaging and superimposing “A”, “B”, “C”, and “D” segments of a single subject. (6) Average superimposed wave forms of “A”, “B”, “C”, and “D” segments for all subjects. Next, the EEG data averaged over all subjects in step 6 were selected for analysis. According to the wave forms of each electrode and the distribution of electrodes in the main time window, the most significant changes in the amplitude of FPZ, F7, F4, F8, C4, P7, P3, and O1 electrodes were selected for further statistical analysis, and the wave forms of the eight electrodes are shown in Figure 8.

#### 3.2.1. Statistical Analysis of Electrode Amplitude

The amplitude changes of human brain waves can reflect the sensitivity of the human body to external environmental influences. In this paper, we first compare the wave amplitude changes of 8 electrodes when users use different apps to complete their tasks, and Table 5 shows the average wave amplitude statistics of each electrode under different APP interfaces for users’ usage behaviors. Then do the repeated measures ANOVA of 4 (different mobile apps: C-Life Senior Care App; Senior Living APP; Senior Care Manager APP; Smart Aging APP) × 8 (electrodes: FPZ, F7, F4, F8, C4, P7, P3, O1).

Firstly, the sphericity test was conducted as shown in Table 6, and the test result was significant *p* = 0.000 < 0.05, which did not satisfy the assumption of spherical distribution and required multivariate ANOVA. After multivariate ANOVA, as shown in Table 7 and Table 8, the results showed that (1) presenting no significant main effect of different mobile application types, *p* = 0.722 > 0.05, indicating no significant difference between mobile applications; presenting a significant main effect of electrode type, *p* = 0.000 < 0.05, indicating a significant difference between electrodes; and (2) presenting a direct electrode and different mobile applications with no significant interaction effect, *p* = 0.425 > 0.05, indicating that there is no interaction between electrodes and different mobile application changes, and the role of electrode factors does not vary with the design elements of mobile applications.

**Figure 9:**
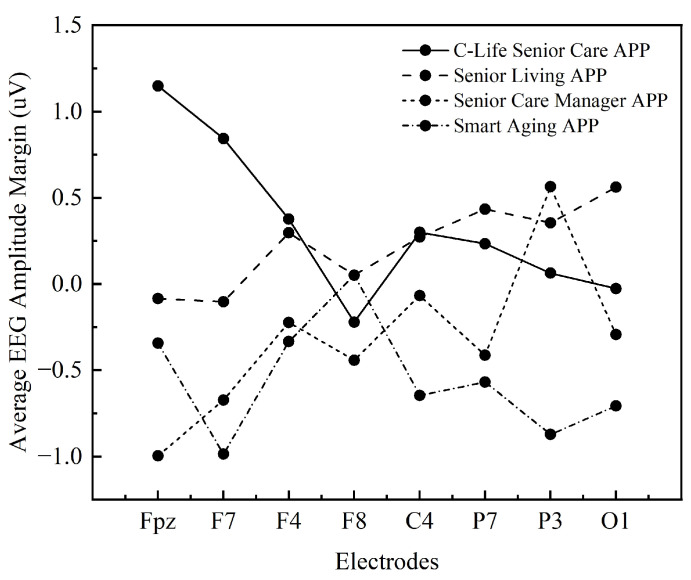
Marginal mean value of electrode amplitude.

Figure 9 visualizes the trend of amplitude change with electrode change. The four apps with different design features have different trends of change with electrodes, among which two types of apps, C-Life Senior Care and Smart Aging, have an overall downward trend of change, and two types of apps, Senior Living and Senior Care Manager, have an overall upward trend of change. In addition, among the four Apps, only C-Life Senior Care APP changes have 1 inflection point, while the other three Apps have 5 inflection points of margin changes, which indicates that the overall changes of C-Life Senior Care APP electrodes tend to be smooth. The largest magnitude and the most obvious magnitude difference existed at electrode Fpz, and the least obvious magnitude difference existed at electrode F8. The overall magnitude size of the four apps was shown as: Senior Care Manager APP > C-Life Senior Care APP > Smart Aging APP > Senior Living APP.

#### 3.2.2. Statistical Analysis of Brain Area Amplitude

The above eight electrodes were divided into four regions: Frontal lobe area (F4, Fpz, F7, F8), Parietal area (P3, P7), Temporal lobe area (C4), and Occipital area (O1), and several electrodes within each region were superimposed and averaged. Table 9 shows the average wave amplitude statistics of each brain area performed by the user under different APP use behaviors, analyzing the differences in statistics of different brain area locations: do 4 (different APP: C-Life Senior Care App; Senior Living APP; Senior Care Manager APP; Smart Aging APP) × 4 (brain area: Frontal area; Parietal area; Temporal area; Occipital area) for repeated measures ANOVA.

The sphericity test was first performed as shown in Table 10, and the test result was significant *p* = 0.000 < 0.05, which did not satisfy the assumption of spherical distribution and required multivariate ANOVA. After multivariate ANOVA, as shown in Table 11 and Table 12, the results showed that there was no significant main effect between the different mobile applications presented, *p* = 0.665 > 0.05, indicating no significant difference between the four mobile application types; there was a significant main effect between the brain regions presented, *p* = 0.000 < 0.05, indicating a significant difference between the brain regions; there was no significant interaction between the brain regions and the different mobile applications presented There is no significant interaction effect between brain regions and different mobile applications, *p* = 0.918 > 0.05, indicating that there is no interaction between brain regions and different mobile applications, and the role of brain region factors does not vary with the design elements of mobile applications.

**Figure 10:**
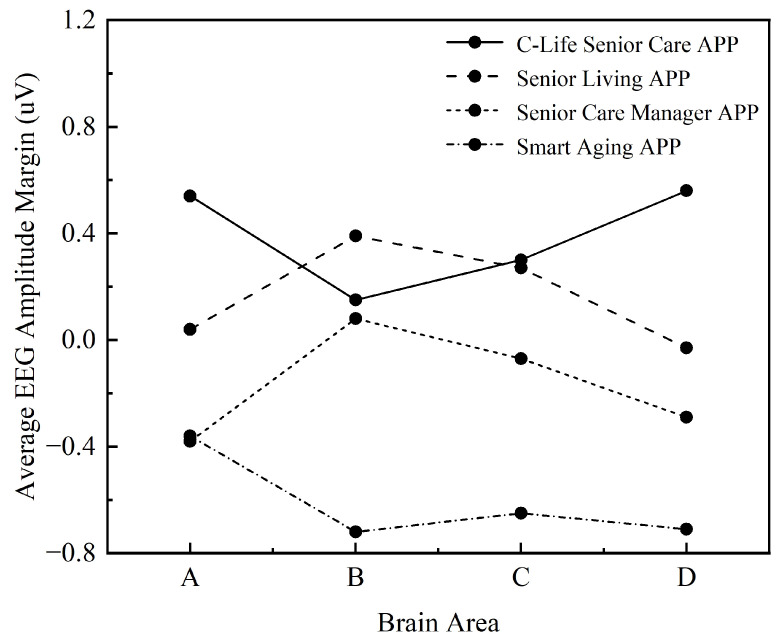
Marginal mean of brain area amplitudes: A refers to Frontal area; B refers to Parietal area; C refers to Temporal area; D refers to Occipital area.

Figure 10 visualizes the trend of the wave amplitude with the change of brain areas. The four apps with different design features have slightly different trends in brain area change. The overall trend direction of Senior Living APP and Senior Care Manager APP is basically similar, with the two categories of C-Life Senior Care and Senior Care Manager having an overall upward trend of change. C-Life Senior Care and Senior Care Manager apps are trending up, while Senior Living and Smart Aging apps are trending down. In addition, among the four apps, only the Smart Aging APP had 2 inflection points, while the other three apps had only 1 inflection point for the margin change, which indicates that the overall brain area change of the four apps tends to be stable. The Occipital area in the brain area had the largest magnitude and the most obvious magnitude difference, and the overall magnitude size of the four apps was shown as follows: Smart Aging APP > C-Life Senior Care APP > Senior Care Manager APP > Senior Living APP.

## 4. Discussion

Cell phones have become a vital tool for human interaction, bringing individuals easy experiences thanks to the ongoing upgrade of 5G communication technology on the Internet. It is vital to make the interface design of a senior-centered mobile application for senior care services user-friendly in order to incorporate older individuals into the online society more quickly [56,57]. This study looked at the influence of different interface design features on user task execution by limiting subjects to simply doing the interface login search task. Additionally, these results demonstrate that varied interface design components have a considerable impact on older users’ ability to accomplish tasks, and both eye-movement and EEG data provide a factual foundation for this assertion.

In terms of the design element of the login screen layout, the time taken to complete the task was much shorter in the hysteretic layout compared to the multi-column and irregular layouts, and the average fixation duration was also very short, with little change in pupil diameter. The irregular and multi-column layouts do not do a good job of attracting the user’s attention, which leads to a tendency for older users to be distracted by information other than the login, so the older population prefers a simpler interface. It is also clear that physiological indicators such as pupil diameter and average fixation duration have a definite advantage in examining the cognitive load of users. This is in line with the findings of other scholars. In terms of the color scheme of the login screen, the fact that the background color of the C-Life Senior Care and Senior Living apps is mostly white makes it easier for the user to perform tasks in a cyclical manner, as the color of the login screen is more appealing to the user than the absence of color. As a consequence, bright design elements should be employed throughout the interface, preferably in crucial information places [58,59,60,61]. The user’s behavioral line of sight can be captured with great accuracy using eye tracking and eye-tracking hotspot maps.

Then, on the element of information density of the login interface, the information density not only refers to the amount of interface information but also refers to whether the logic between the information is smooth [62]. When older adults completed the login task in the Senior Living APP, for example, the magnitude of both electrodes and brain areas was the smallest compared to the other three apps, indicating that an interface design with too much information density and not enough logical arrangement of information will cause older adults to lose interest in the process of using it. It has also been verified that changes in brain areas and electrode changes can reflect changes in user behavior and emotions. Finally, in terms of the element characteristics of the login icon, because the login symbol is the most essential and key information point in the login interface, its position, size, and design should correspond to the visual habits of older users. The user’s pupil diameter did not change greatly during the task execution process in the Senior Care Manager APP and Smart Aging APP, indicating that there is no significant cognitive load on information acquisition during task execution and that the central position of the login icon in the interface is beneficial for older people.

The interface layout and the reasonable design of overall information (including the contrast between the background color and the font/icon color, the position of the login symbol in the body of the interface, the size of the login icon in the body of the interface, and the overall information density of the interface, etc.) can guide the visual focus and browsing habits of users, thus affecting the task execution of user behavior, according to the analysis of users’ eye movement and EEG data during the use of four typical pension service mobile applications. This study evaluates the variations between four popular mobile applications for social services for older persons and identifies the interactive impacts of key interface design components on the impact of task performance on older individuals. The study’s findings, which combine objective experimental findings with subjective user interaction, offer a theoretical framework for future interface designs that are centered on the needs of older people and suggest the following design tactics: (1) If task performance for older users is the only factor taken into account, then a palatial interface style with minimal information density, a high percentage of login icons, and moderate placement is chosen; an irregular interface layout must reduce information density, eliminate distracting icons, and emphasize login icon elements in order to increase older people’s motivation to perform tasks. A multi-column interface layout with low information density and a moderate proportion of login icons is more motivating for older people. (2) No matter how the interface is set up, the login symbol is more noticeable than other components (be it by placing larger icons, adding color, repositioning, etc.) and the user interaction experience benefits from the lower information density level. (3) Focus on adjusting the information density of the interface if you want to increase customer satisfaction with the product. Only essential information can be retained in a low-density interface, while a high-density interface level can diversify high-density information through the use of colors, larger and bolded fonts, and other techniques to highlight significant information. (4) Based on a study of the visual behavior of users using the interface, it can be found that at the level of visual centrality of the login icon and low density interface, the rational use of color can reduce the time users spend searching for the login button and improve the efficiency of task execution.

## 5. Limitations and Future Directions

There are some limitations to this study. First, this study used different applications as independent variables, and although it can draw conclusions about the influence of design elements like interface layout, interface color, and interface information density on the use of APP by older adults, it may not yield as precise results as single element variable experiments such as control chart-book layout; second, the sample size of male and female control groups of subjects was uneven. In future studies, we should make sure that the experimental independent variables are not influenced by other strong variables and average the number of control groups.

## 6. Conclusions

With the inclusion of the new crown pandemic, increasing the frequency of social encounters for older individuals using smart gadgets could help them feel less lonely and improve their physical health. On the other hand, older persons have a high demand for the ability to precisely and efficiently detect the interaction information of mobile applications so that they may simply operate the program. In this work, we investigate the effects of multidimensional design features of interfaces on task performance in the elderly using a mix of eye-movement and EEG tests. The findings of these trials suggest that the most beneficial combination of interface design elements for task execution for older people is a palatial interface layout, a large proportion of login icons, a small information density level, and effective use of color placement. It was also further demonstrated that changes in pupil diameter, mean gaze duration, and trajectory heat maps in the eye movement data and changes in potential and brain region amplitude in the EEG signal clearly reflect the true visual cognitive state of the user during use. The palatial interface architecture organizes the information on the interface and makes it easier for older persons to operate. The proportion of login icons in the interface and the richness of interface information have an impact on the old person’s task performance when using the app. A higher proportion of login icons and a lower degree of information density are more ideal for older persons to use and make it easier to assist them through the interaction process to properly obtain and process information. Furthermore, the use of color in the interface and the display of vital information will make it easier for older people to use. It was also further demonstrated that changes in pupil diameter, mean gaze duration and trajectory hotspot maps in the eye movement data, and changes in potential and brain region amplitude in the EEG signal clearly reflect the true visual cognitive state of the user during use.

Based on two objective evaluation criteria, namely eye movements and EEG, with specific theoretical and practical values, this study explores the impact of various interface design features on older people’s ability to conduct task-related activities. In terms of theoretical significance, this work adds to the body of knowledge regarding interface design for older users by applying a combined eye-movement and EEG method to the assessment of the effects of interface design features on task performance. Secondly, this study demonstrates that user behavioral and emotional changes can be observed using data markers connected to eye movements and EEG signals. Indicators indicate that older people perceive information on the interface slowly and feel negative emotions. They include long average fixation duration, significant pupil diameter changes, and minor amplitude changes of electrodes and brain areas in the EEG. This research compares and explains the differences between four typical mobile applications of social services for the older person, which helps designers and other professionals comprehend the impact of each design element on the initial interface design and later software upgrade process. The effects of task completion offer strategic direction for the creation of interface design products geared toward older persons, which can help older persons continue to use smart device software.

## Figures and Tables

**Table 1:** Basic participant information statistics table.

	Participants	
	Male	Female
Number	7	9
Mean ± SD	60.57 ± 6.58	62.33 ± 8.28

**Table 2:** ANOVA of participants’ task completion time for different Apps: A refers to C-life Senior Care APP; B refers to Senior Living APP; C refers to Senior Care Manager APP; D refers to Smart Aging APP.

	Applications								ANOVA	
	Group A (n = 16)		Group B (n = 16)		Group C (n = 16)		Group D (n = 16)			
	Mean	SD	Mean	SD	Mean	SD	Mean	SD	F	P
Task Completion time (s)	25.50	15.81	24.44	21.58	20.55	18.21	21.23	12.34	3.240	0.093

**Table 3:** ANOVA of participants’ average fixation duration for different Apps: A refers to C-life Senior Care APP; B refers to Senior Living APP; C refers to Senior Care Manager APP; D refers to Smart Aging APP.

	Applications								ANOVA	
	Group A (n = 16)		Group B (n = 16)		Group C (n = 16)		Group D (n = 16)			
	Mean	SD	Mean	SD	Mean	SD	Mean	SD	F	P
Fixation duration (s)	6.39	6.33	7.36	9.11	6.47	6.42	4.84	5.97	1.667	0.007

**Table 4:** ANOVA of participants’ mean pupil diameter for different Apps: A refers to C-life Senior Care APP; B refers to Senior Living APP; C refers to Senior Care Manager APP; D refers to Smart Aging APP.

	Applications								ANOVA	
	Group A (n = 16)		Group B (n = 16)		Group C (n = 16)		Group D (n = 16)			
	Mean	SD	Mean	SD	Mean	SD	Mean	SD	F	P
Mean Pupli diameter (mm)	3.04	0.44	2.46	1.28	2.82	0.84	2.67	1.14	0.931	0.008

**Table 5:** Analysis of the mean value of the amplitude of each electrode: A refers to C-life Senior Care APP; B refers to Senior Living APP; C refers to Senior Care Manager APP; D refers to Smart Aging APP.

Electrodes		Mean	SD	Number	Electrodes		Mean	SD	Number
Fpz	A	0.004665	27.31288	16	F7	A	−0.02338	20.20405	16
	B	0.134871	21.20736	16		B	0.093055	16.96126	16
	C	0.474146	26.35667	16		C	0.857294	23.63811	16
	D	−0.06103	17.80065	16		D	−0.010486	13.98773	16
	Total	0.138163	23.16939	64		Total	0.916483	18.69779	64
**Electrodes**		**Mean**	**SD**	**Number**	**Electrodes**		**Mean**	**SD**	**Number**
F4	A	−0.02202	14.76019	16	F8	A	−0.03429	12.74355	16
	B	0.113233	13.24345	16		B	0.108794	12.79345	16
	C	0.538435	23.20325	16		C	0.708056	19.00214	16
	D	−0.02329	11.67699	16		D	0.040016	10.13491	16
	Total	0.15159	15.72097	64		Total	0.205644	13.66815	64
**Electrodes**		**Mean**	**SD**	**Number**	**Electrodes**		**Mean**	**SD**	**Number**
C4	A	−0.01727	11.89204	16	P7	A	−0.01281	12.46189	16
	B	0.060629	10.98026	16		B	0.051005	14.20482	16
	C	0.424661	15.35449	16		C	0.381196	22.86642	16
	D	−0.01441	10.28045	16		D	−0.05949	12.55813	16
	Total	0.113403	12.12681	64		Total	0.089975	15.52282	64
**Electrodes**		**Mean**	**SD**	**Number**	**Electrodes**		**Mean**	**SD**	**Number**
P3	A	−0.01503	17.14472	16	O1	A	−0.03526	18.31962	16
	B	0.05162	12.82255	16		B	0.0478	16.6618	16
	C	0.351402	17.40791	16		C	0.401782	17.03462	16
	D	−0.07309	11.5843	16		D	−0.08066	12.17158	16
	Total	0.078726	14.73987	64		Total	0.083416	16.04691	64

**Table 6:** Mauchly sphericity test.

Within-Subject Effects	Mauchly	Approximate Chi-Square	Degrees of Freedom	P	Epsilon ${}^{b}$		
					Greenhouse- Geisser	Cyn Feldt	Lower Limit
brain area	0.164	3694.900	27	0.000	0.664	0.666	0.143

^*b*^ May be used to adjust the degrees of freedom for the averaged tests of significance. Corrected tests are displayed in the Tests of Within-Subjects Effects table.

**Table 7:** ANOVA for different applications.

	Class III Sum of Squares	Degrees of Freedom	Mean Square	F	P
Intercept	34.563	1	34.563	0.023	0.880
Different Apps	2002.538	3	667.513	0.443	0.722
Errors	3,085,338.250	2048	1506.513		

**Table 8:** ANOVA for electrode × APP.

		Value	F	Assumption Degrees of Freedom	Error Degrees of Freedom	P
Intercept	Billy trajectory	0.002	0.618 ${}^{b}$	7.000	2042.000	0.000
	Wilke Lambda	0.998	0.618 ${}^{b}$	7.000	2042.000	0.000
	Hotelling track	0.002	0.618 ${}^{b}$	7.000	2042.000	0.000
	Roy Max Root	0.002	0.618 ${}^{b}$	7.000	2042.000	0.000
Intercept × Apps	Billy trajectory	0.011	1.027	21.000	6132.000	0.425
	Wilke Lambda	0.990	1.027	21.000	5864.072	0.425
	Hotelling track	0.011	1.027	21.000	6122.000	0.426
	Roy Max Root	0.005	1.466 ${}^{c}$	7.000	2044.000	0.175

^*b*^ Exact statistic. ^*c*^ This statistic is the upper limit of F that generates the lower limit of significance level.

**Table 9:** Analysis of the mean amplitude of each brain region.

Brain Area		Mean	SD	Number
Frontal Lobe Area	C-Life Senior Care APP	0.5363	10.49246	16
	Senior Living APP	0.0364	17.20792	16
	Senior Care Manager APP	−0.3559	19.38743	16
	Smart Aging APP	−0.3800	10.83575	16
	Total	−0.408	14.98940	64
**Brain Area**		**Mean**	**SD**	**Number**
Parietal Area	C-Life Senior Care APP	0.1480	10.28221	16
	Senior Living APP	0.3940	16.71846	16
	Senior Care Manager APP	0.0752	21.89155	16
	Smart Aging APP	−0.752	13.09230	16
	Total	0.0258	16.08620	64
**Brain Area**		**Mean**	**SD**	**Number**
Temporal Lobe Area	C-Life Senior Care APP	0.2992	8.87934	16
	Senior Living APP	0.2720	15.38968	16
	Senior Care Manager APP	−0.0673	13.48589	16
	Smart Aging APP	−0.6464	10.60769	16
	Total	0.0356	12.34672	64
**Brain Area**		**Mean**	**SD**	**Number**
Occipital Area	C-Life Senior Care APP	−0.0270	15.71846	16
	Senior Living APP	0.5609	19.61370	16
	Senior Care Manager APP	0.2936	17.44823	16
	Smart Aging APP	−0.7069	12.71110	16
	Total	0.1166	16.56066	64

**Table 10:** Mauchly sphericity test.

Within-Subject Effects	Mauchly	Approximate Chi-Square	Degrees of Freedom	P	Epsilon ${}^{b}$		
					Greenhouse- Geisser	Cyn Feldt	Lower Limit
brain area	0.670	820.670	14	0.000	0.437	0.463	0.200

^*b*^ May be used to adjust the degrees of freedom for the averaged tests of significance. Corrected tests are displayed in the Tests of Within-Subjects Effects table.

**Table 11:** ANOVA for different applications.

	Class III Sum of Squares	Degrees of Freedom	Mean Square	F	*p*
Intercept	24.581	1	24.581	0.035	0.857
Different Apps	1122.500	3	374.167	0.526	0.665
Errors	1,457,707.584	2048	711.711		

**Table 12:** ANOVA for brain region × APP.

		Value	F	Assumption Degrees of Freedom	Error Degrees of Freedom	*p*
Intercept	Billy trajectory	0.000	0.044 ${}^{b}$	3.000	2046.000	0.000
	Wilke Lambda	1.000	0.044 ${}^{b}$	3.000	2046.000	0.000
	Hotelling track	0.000	0.044 ${}^{b}$	3.000	2046.000	0.000
	Roy Max Root	0.000	0.044 ${}^{b}$	3.000	2046.000	0.000
Intercept × Apps	Billy trajectory	0.002	0.434	9.000	6144.000	0.918
	Wilke Lambda	0.998	0.434	9.000	4969.577	0.918
	Hotelling track	0.002	0.433	9.000	6134.000	0.918
	Roy Max Root	0.002	1.057 ${}^{c}$	3.000	2048.000	0.366

^*b*^ Exact statistic. ^*c*^ This statistic is the upper limit of F that generates the lower limit of significance level.

## Data Availability

Not applicable.

## Abbreviations

The following abbreviations are used in this manuscript:

APP	Application
ECG	Electrocardiography
EMG	Electromyography
EEG	Electroencephalography
